# Special Issue “MicroRNAs and Other Non-Coding RNAs as Regulators, Biomarkers, and Therapeutic Targets, 2nd Edition”

**DOI:** 10.3390/ijms27188392

**Published:** 2026-09-20

**Authors:** Wan Lee

**Affiliations:** 1 Department of Biochemistry, Dongguk University College of Medicine, 123 Dongdae-ro, Gyeongju 38066, Republic of Korea; wanlee@dongguk.ac.kr; Tel.: +82-54-770-2409; 2 Section of Molecular and Cellular Medicine, Medical Institute of Dongguk University, Gyeongju 38066, Republic of Korea

## 1. Introduction

Two decades of work have produced many thousands of reported associations between individual non-coding RNAs and human disease, yet only a very small number have entered clinical use. The distance between those two quantities is usually attributed to translation difficulties, costs, or regulatory burdens. A more immediate obstacle is visible in the primary literature itself. For a large proportion of the microRNAs that have been proposed as biomarkers, it is possible to find one study reporting the molecule elevated in a given disease and another, of comparable quality, reporting it reduced in the same disease.

The customary responses are to attribute such disagreements to chance, to small sample sizes, or to publication bias, and to wait for a larger study to settle the matter. Larger studies have not settled this. A different reading is proposed here. The direction of a reported change is not a property of the microRNA. It is a property of a measurement made in a particular compartment, in a particular population, at a particular disease stage, on a particular platform, against a particular normalizer. Stated in the customary form, that miR-X is upregulated in disease Y, and a result omits every term that determines it, which is why it fails to replicate for reasons that have little to do with biology.

Five recent papers illustrate both the problem and a workable response to it. Four are original studies, and one is a review, all published between 2024 and 2026. They range from the hair follicle of a Chinese sheep breed to the subcutaneous adipose tissue of adults with obesity, the serum of pregnant South African and Spanish women, the plasma of Mexican children, and the mechanosensory apparatus of skeletal muscle. Superficially, they share only a class of molecule. What they also share is that every one of them, at some point in its discussion, is obliged to account for a published result that points the other way.

## 2. Discordance Is the Rule, Not the Exception

These five papers are the contributions to the Special Issue “MicroRNAs and Other Non-Coding RNAs as Regulators, Biomarkers, and Therapeutic Targets, 2nd Edition”, which now closes. Their separate confrontations with the prior literature are worth setting out in some detail [contributions 1–5].

Masete et al. [contribution 1] report lower circulating miR-20a-5p in South African women with gestational diabetes mellitus. This replicates an earlier cohort from the same group [1] and contradicts two independent Chinese cohorts in which the same microRNA was elevated [2,3]. The authors do not smooth this over. They set out the plausible sources of the disagreement, namely sample compartment, amplification chemistry, and, above all, the choice of normalizer, endogenous miR-221 in one study against exogenous spike-in controls in another.

Contreras-Ramos et al. [contribution 2] find let-7a-5p reduced in the plasma of Mexican children with obesity and state plainly that this contradicts Krause et al. [4], who reported the same microRNA increased in children of comparable age carrying features of metabolic syndrome. They attribute the divergence to genetic, environmental, and sociocultural differences between the study populations.

Markina et al. [contribution 3] found that miR-142 was decreased in subcutaneous adipose tissue in obesity, whereas their earlier work found it increased in the circulation of the same disease [5]. A large circulating study reported a steep rise associated with insulin resistance and systemic inflammation [6]. They conclude that transcription and degradation of this microRNA proceed at different rates in the two compartments, so that a tissue measurement and a plasma measurement are not two views of one quantity.

Three papers, three continents, three independent contradictions, each acknowledged rather than buried. This is not a defect peculiar to these studies. It is the ordinary condition of the field.

## 3. What Follows from This, and What Does Not

The tempting inference is that circulating non-coding RNA measurements are too unstable to be worth pursuing. The evidence assembled here argues the opposite. Each of these disagreements dissolves once the measurement is fully specified, and the terms that need specifying are known and finite [7,8]. Two practical consequences follow, and both are already visible in the contributions themselves.

## 4. Stratify Within the Diagnosis Rather than Against Health

Markina et al. [contribution 3] compare metabolically healthy obese individuals with metabolically unhealthy obese individuals rather than obese individuals with lean controls, and the outcome is instructive. The inflammation markers CCL2 and miR-155 are elevated in both phenotypes, making them poor discriminators despite being highly significant compared with controls. HIF1A and miR-378 rise only in the unhealthy phenotype, and the suppression of the antifibrogenic miR-142 is deepest there. The markers that separate disease from health and those that separate a stable from a progressing phenotype are not the same.

Masete et al. [contribution 1] reach the mirror image from the opposite direction. Profiling type 1, type 2, and gestational diabetes during pregnancy, they find that the microRNA signatures are largely shared across all three, with only small quantitative differences, and conclude that the degree of hyperglycemia rather than the etiology shapes the profile, consistent with an earlier cross-diabetes comparison [9]. Both papers point to the same underlying problem. Diagnostic labels are not the natural units of the molecular variation.

## 5. Combination Rather than Substitution

Neither group presents its microRNAs as standalone tests, and their own numbers explain why. In Contreras-Ramos et al. [contribution 2], miR-126-3p alone yields an area under the curve of 0.39, i.e., no discriminatory value whatsoever, whereas the same measurement combined with body mass index percentile and the antioxidant enzymes superoxide dismutase and glutathione peroxidase yields 0.97. Alone, miR-16-5p gives 0.59 and rises to 0.94 once triacylglycerol and insulin are added. In Masete et al. [contribution 1], miR-20a-5p alone yields 0.69, and this increases to 0.87 with maternal age, gestational age, and body mass index.

Two groups on different continents, working with different populations and platforms, converge on the same conclusion. The clinical value of these molecules lies in the information they provide beyond variables already being measured, not in displacing them. A microRNA that fails as a solitary marker may still be doing real work.

Both papers also show why this requires discipline. In Masete et al. [contribution 1], the validated differences did not survive Bonferroni correction [10], a limitation the authors report rather than omit. In Contreras-Ramos et al. [contribution 2], the four-parameter model was fitted and evaluated in the same one hundred children. Combination models inflate apparent performance unless they are tested on an independent sample, and independent validation should be the default expectation rather than an aspiration in this area.

## 6. The Durable Anchor Is Mechanism

If the direction of an association depends on context, the only stable reference point is a causal chain. He et al. [contribution 4] supply the one complete example in this collection. Beginning from a microRNA differentially expressed between wool phenotypes, they predict GNAI2 as a target, test that prediction with a dual luciferase reporter carrying a seed mutant control, manipulate both the microRNA and the target in gain and loss of function, trace the effect through Wnt and beta-catenin transcriptional activity, and terminate in a cellular phenotype, namely, the proliferation and inductive capacity of dermal papilla cells. It is worth noting where this was done. Ovine dermal papilla cells carry no clinical stakes and therefore impose no pressure to declare a biomarker. The rigor and the modest setting are not unrelated.

Ngo et al. [contribution 5] apply the same discipline to a question where the evidence does not yet exist. Reviewing Piezo1 mechanotransduction alongside the myogenic non-coding RNA program, they decline to assert the coupling that the two literatures invite, and instead state three predictions together with explicit criteria for their failure. Their unbiased target screen is the informative part. It returns six candidate microRNAs for the PIEZO1 untranslated region, none of which is enriched in skeletal muscle, a result that weakens rather than strengthens their own preferred hypothesis and is reported as such. Precedent for such coupling exists in vascular smooth muscle [11] and cardiac fibroblasts [12], but precedent is not evidence. Declaring in advance what would refute a hypothesis remains the cheapest available protection against the accumulation of unfalsifiable claims, a hazard to which the competing endogenous RNA literature is particularly exposed [13,14].

## 7. The Therapeutic Ambition

The third term in the title of this Special Issue, therapeutic targets, is not yet satisfied by any contribution to it, and the argument above indicates why. Ngo et al. [contribution 5] make the point explicitly for Piezo1, where activation is beneficial in disuse atrophy, while inhibition is beneficial in the calcium-overloaded dystrophic fiber, so that the correct intervention is normalization towards a context-specific optimum rather than uniform enhancement or suppression. The same logic transfers directly to a microRNA whose direction of change differs between tissue and plasma, or between a stable and a progressing phenotype. A mimic or an inhibitor administered without knowing which context the patient occupies is as likely to be wrong as right. Demonstrations that delivery of a single non-coding RNA can shift a disease phenotype in vivo already exist [15,16], so the obstacle is not feasibility. It is specification.

## 8. What This Implies for Reporting Practice

Three requests follow from the five papers gathered here, addressed to anyone working in this area. First, report compartment, normalization strategy, platform, population, and disease stage as primary methodological facts rather than as supplementary material, and state the direction of any change with that context attached. Second, submit discordant and negative results. Every contribution to this issue was strengthened by confronting a contradiction, and the contradictions are only informative if they are published. Third, for each proposed mechanism, state what observation would refute it; for each combination model, validate it outside the sample in which it was built.

None of this is novel advice. It is the ordinary practice of experimental biology, applied to a class of molecules whose measurements have proved unusually sensitive to conditions that are easy to overlook. The non-coding RNA field does not have a reproducibility problem so much as a specification problem, and the second is considerably easier to fix.
